# The interaction of HIV-1 and HIV-2 with cellular protein trafficking pathways

**DOI:** 10.1186/1742-4690-10-S1-P2

**Published:** 2013-09-19

**Authors:** Justine Alford, Robert Spooner, Michela Marongiu, Emma Anderson

**Affiliations:** 1 School of Life Sciences, University of Warwick, Coventry, UK

## Background

Assembly and release of HIV-1 particles occurs at the plasma membrane and is dependent on trafficking of Gag from sites of protein synthesis to sites of particle assembly. For example, the interaction of HIV-1 Gag with components of cellular trafficking pathways such as clathrin adaptor proteins (AP) 1 and 3 has been shown to be required for HIV-1 virion production [1,2]. This is likely to place a large burden on cellular trafficking pathways, but this has not previously been investigated. We addressed the questions of whether HIV-1 disrupts cellular protein trafficking and whether HIV-2 has the same effect.

## Materials and methods

We used the well characterised pathways of diphtheria toxin (DTx) and ricin toxin (RTx) trafficking to measure the effect of HIV-1 and HIV-2 on toxin-induced inhibition of protein synthesis. HIV-1 and -2 Gag localisation, and the role of AP-3 in Gag trafficking, was studied using confocal immunofluorescence microscopy, siRNA-mediated knockdown of AP-3, and virion release assays.

## Results

HIV-1 had a protective effect against DTx-, but not RTx-induced cytotoxicity, demonstrating that endosomal trafficking is specifically disrupted in the presence of HIV-1. However, HIV-2 had a significantly higher protective effect against DTx, suggesting greater disruption of endosomal trafficking by HIV-2. In agreement with these results, we observed a much higher proportion of HIV-2 Gag localised to endosomal compartments than HIV-1 Gag. There was strong co-localisation between HIV-2 Gag, AP-3 and clathrin, but less so with HIV-1 Gag. siRNA-mediated knockdown of AP-3 resulted in an increase in the proportion of both HIV-1 and HIV-2 Gag localised to endosomal compartments, a decrease in the proportion at the plasma membrane, and a reduction in virion release.

## Conclusions

HIV-1 and HIV-2 both utilise cellular protein trafficking pathways, which disrupts normal cell functioning. HIV-2 has a particularly disruptive effect on the endosomal pathway, and we observed that HIV-2 Gag accumulates in endosomal compartments. AP-3 and clathrin are recruited to these compartments in the presence of HIV-2, and we hypothesise that this is a requirement for transport of Gag and/or virions from endosomes to the plasma membrane.
